# Factors influencing men’s decisions about a career in nursing

**DOI:** 10.1371/journal.pone.0337360

**Published:** 2025-12-05

**Authors:** Angela V. Flynn, Oisín Brenock, Mohammad M. Saab

**Affiliations:** 1 School of Nursing and Midwifery, University College Cork, Cork, Ireland; 2 South Infirmary Victoria University Hospital, Cork, Ireland; University of Sharjah College of Health Sciences, UNITED ARAB EMIRATES

## Abstract

**Background:**

Despite many efforts to diversify the nursing workforce, in most countries the profession remains predominantly feminine. It is widely accepted that with a greater gender balance, nursing would be better understood, would provide more holistic care, may achieve greater professional acknowledgement, and may achieve higher levels of remuneration. To address this imbalance, it is worth uncovering the factors which influence the career decision making of young men.

**Objectives:**

To capture the views of men in nursing and explore the opinions of secondary school students to understand their decision-making regarding nursing as a career choice, and the factors that influence those decisions.

**Design:**

Cross-sectional survey.

**Setting:**

Recruitment was primarily through social media and e-mails to students, graduates, colleagues, and professional nursing and academic networks.

**Participants:**

Convenience and snowball sampling strategies were used to recruit men aged 18 or above in secondary schools (Cohort 1), and men aged 18 or above who were already employed as nurses or were undergoing a nurse education programme (Cohort 2).

**Methods:**

Online surveys comprising Likert scale and open-ended questions were completed. Quantitative data were analysed using descriptive statistics. Qualitative data were used to support and add depth to the quantitative findings. Illustrative participant excerpts were used to support the findings.

**Results:**

A total of 130 surveys were completed. Our findings show that factors such as gender-based stereotypes and portrayals of nursing in media, are the least influencing factors that impacted on men choosing a career in nursing. In fact, the two most influential factors were found to be contact with nurses and experience with healthcare related activities such as volunteering/first aid.

**Conclusions:**

Findings illustrate the influential role that all nurses have in encouraging men into a career in nursing. The findings also have implications for the allocation of resources towards recruitment and career information strategies.

## Introduction

Modern healthcare providers and employers strive to provide a dynamic and inclusive workplace for a wide variety of healthcare professionals. Working in healthcare usually guarantees a diverse range of colleagues from differing walks of life, countries, religions, and genders [1,2]. Despite a broad welcome for diversity in healthcare, nursing as a profession remains gendered with women vastly outnumbering men, who tend to make up only approximately 10% of students entering undergraduate nurse education programmes [3]. Some improvements have been noted in recent years, however globally nursing remains 85% female [2]. This ought to be addressed further to ensure a nursing workforce that reflects the diversity of the patient population it serves [4]. Decisions to choose a nurse education course and career are subject to several influencing factors. The current study allows us to hear from young men currently making career choices and others who have already chosen a career in nursing. We can thereby understand better how these two cohorts report the impact of various factors.

The literature illustrates a very wide range of studies such as those detailing perceived barriers met when recruiting men into nursing [5]. Some papers have examined the impact on the profession as a whole [6], while others have reported a strong demand for male nurses from patients [6]. Some have proposed solutions such as graduate entry programmes [7], while more recently the global shortage of nurses [8] has been cited as a further argument for the recruitment of more men into nursing [9].

Many health service and higher education organisations invest considerable time and effort into specific recruitment campaigns to encourage a diverse range of applicants [10]. Although, it is sometimes unclear the extent to which these make an impact. For example, assumptions may be made about the extent to which young people making career choices are influenced by their career guidance advice, by family experiences, by media portrayals, and/or other factors. In order to be in a position to create and invest in appropriate strategies to address the gender imbalance in nursing, a greater understanding of the factors affecting men’s choices regarding the profession is needed [11].

## Background literature

The literature in relation to men in nursing was reviewed. A search in CINAHL and PubMed was conducted using the following keywords: Men in nursing, gender stereotypes, challenges, barriers, and lived experience. Studies published within a 10-year timeframe were sought. A double screening process was undertaken by two authors. A total of 22 potentially relevant studies were identified. Of those, 15 relevant studies were reviewed and included to provide the evidence base for the current study.

There exists a wide range of studies focused on the lived experiences of men in the nursing profession, and of male nursing students’ experience in college [12,13]. There were some common themes and findings that arose, such as men seemingly feeling drawn to nursing through previous experience they had, such as experiencing nursing care as a patient themselves, having a family member in care, or previous work experience [14]. It has also been suggested that having a close family member who is a nurse was also a common influence for men to enter nursing [11].

Many men did not consider a career in nursing until around the time that they were finishing secondary education at around the age of 18–19 years [11]. Many also turned to nursing after having previously worked in other fields or in other careers [12]. While men in the reviewed studies reported feeling proud to be working as nurses or studying to become a nurse, many seemingly felt reluctant to mention their job title or college course in social situations out of fear of ridicule or gender stereotyping. Almost all the men in these papers had experienced some form of stereotyping because of their chosen profession. In most cases, this was some form of homosexual stereotyping [15].

Several men cast light on how the professional status of nursing can often be a major factor in dissuading many men from entering into the profession [16]. Many felt that the public had a skewed perception of what nursing actually entails, which was seemingly not helped by portrayals of nursing in the media, especially that of men in nursing [15]. As a result, men felt that nursing was often looked upon as “lower” or “lesser” than other healthcare professions in the eyes of the public. Consequently, many men experienced family members dissuading them from choosing nursing or telling them that they could use it as a stepping-stone in order to go on to study medicine.

Many men reported feeling a sense of isolation within the profession due to some differences between men and women, such as communication [12]. Men said they tended to use more direct communication which could be perceived to be hostile by some of their female colleagues [13]. Some complained of feeling excluded, almost like an “outsider within.” Other reasons for this sense of isolation stemmed from having no male role models to look up to within the profession [12].

Three studies spoke of how more could be done within secondary schools to promote nursing to male students. This was because in some of the studies, men spoke of little support or information in secondary school when it came to deciding to choose to study nursing [11,13]. Recommendations to promote nursing to male students more, included having male speakers talk to students during career open days or school visits, as many men felt they had no male role models within the profession [11,17].

The maternity setting frequently came up in the reviewed studies as having been a very negative experience in terms of clinical placement [13]. Many men felt they were viewed negatively and got little to no patient contact as a result. They felt confused as to why patients in the maternity setting had no issue with a male doctor visiting them, but the male nursing students were frequently turned away. Overall, they felt unwelcomed in this setting which hindered their learning [13,18].

Some male nursing students spoke of some issues they had experienced within the college setting [15]. Many of the men had experienced educational instructors who they felt tended to pick on them more in the classroom. Others felt that this was because they tended to question the instructors more which led to them being picked on more [18]. Whilst attending lectures, students spoke of lecturers and teachers consistently referring to the nurse as “she” or “her,” which led to a feeling of alienation or loneliness within the classroom; this continued into assignments and exam questions [19]. Many talked about the strange feeling of being recognised very early on in the course, simply because they were one of very few male students. Although this was not necessarily seen as a negative experience, some men felt alienated as a result [20].

Overall, men wanted to alter the view of nursing as specifically a woman’s career [11]. The term “male nurse” was a topic of particular interest, with many having strong feelings against the term, as they felt that it differentiated them from their colleagues, which they did not favour. Instead, they preferred the term “nurse” as they were of the view that they are no different from their female colleagues [11].

Our overview of the literature revealed many common themes surrounding men in nursing, as well as men studying nursing, with many challenging and negative experiences reported. There appears to be few studies looking at students in secondary schools. It would appear that so as to invest appropriately in strategies to address the gender imbalance in nursing, we should have a much clearer understanding of what it is that influences men’s decision-making regarding a career in nursing. Therefore, this study aimed to capture the views of men in nursing and explore the opinions of secondary school students to understand their decision-making regarding nursing as a career choice, and the factors that influence those decisions.

### Methods

This study used a cross-sectional survey design and data collection took place between 25/07/2021 and 25/09/2021. No theoretical underpinning was used since the aim of the study was to obtain candid and unadorned responses from participants without necessarily adhering to pre-existing theories or views of the topic under study.

### Participants

Non-probability convenience sampling was used to recruit two participant cohorts. Cohort 1 was made up of men aged 18 or above in secondary schools and Cohort 2 consisted of men aged 18 or above who were already employed as nurses or were studying to become a nurse. Snowball sampling was also used whereby participants who completed the survey were encouraged to share the survey link with their colleagues.

### Instruments

Online surveys of two cohorts of participants were undertaken allowing a wide distribution within a short timeframe. The survey questions were derived from their repeated prominence within the reviewed literature (Table 1). The survey was then customised for each of the two Cohorts. The survey for Cohort 1 comprised 20 items. Of those, 15 were rated on a Likert scale ranging from 1 star for “Very Discouraging” to 5 stars for “Very Encouraging.” The remaining five questions were open-ended, exploring other influencing factors in participants’ career choice as well as family and friends’ reactions to participants choosing a career in nursing.

**Table 1:** Factors featured in the literature, used to develop the survey items.

Survey item	Corresponding citation
Family members	Harding et al. (2018) Zamanzadeh et al. (2013)
Career guidance advice	Wilson (2005) Whitford et al. (2020)
Information from open days	Wilson (2005) Whitford et al. (2020)
College websites	Pool (2012)
Contact with nurses	McLaughlin et al. (2010) Harding et al. (2018)
Portrayals of nursing in media (TV/film)	Meadus and Twomey (2011)
Involvement in voluntary healthcare activities	Harding et al. (2018)
Peers’ opinions	McLaughlin et al. (2010) Zamanzadeh et al. (2013)
Personal research	Pool (2012)
Awareness of demand for nurses	World Health Organization (2025) International Council of Nurses (2021)
Portrayal of nursing during COVID-19	Dos Santos (2020)
Social media	Meadus and Twomey (2011)
Gender-based stereotypes	O’Connor (2015)

The survey for Cohort 2 comprised 19 items. Of those, 13 were rated on a Likert scale ranging from 1 star for “Very Discouraging” to 5 stars for “Very Encouraging.” The remaining six questions were open-ended, exploring other influencing factors in participants’ career choice as well as family and friends’ reactions to participants choosing a career in nursing. The survey was piloted with six participants and no changes were made. Therefore, responses from these participants were included in the final analysis. The full survey can be found in Supporting Information File 1.

### Data collection and analysis

Recruitment was primarily via social media (i.e., Twitter, Facebook, and Instagram) as well as via emails to students, graduates, colleagues, and professional nursing and academic networks. Links to the two online surveys were widely shared on social media and via e-mail between June and August 2021. As a result, the number of men contacted is unknown. While it is difficult to estimate the total reach of the links to the survey, the posts on social media had a moderate impact when evaluated through the total number of impressions and engagements over the course of four posts (Table 2). Impressions are defined as the times that the post is seen on the platform, whereas engagements are the total number of times a user interacts with a post. This includes all clicks anywhere on the post, reposts, replies, follows, and likes.

**Table 2:** Total reach, impressions, and engagements over four social media posts.

	Reach	Impressions	Engagements
**Twitter**	–	33,931	607
**Instagram**	–	2,016	21
**Facebook**	4,430	–	129

To ensure that only eligible participants completed the survey, we included an introductory paragraph within the survey explaining the purpose of the study and eligibility criteria. The survey was open for responses from July to August 2021. A detailed information section was provided at the start of the survey to ensure informed consent. Participants were then required to give their written consent to participate and confirm that they were aged 18 years and above in order to continue to the following section. No major ethical issues were anticipated. Ethical approval was gained from the Social Research Ethics Committee at University College Cork, Ireland. Data were stored on password protected computers within a secure cloud-based storage system.

Data from the online surveys were exported to Microsoft Excel. Quantitative survey data were analysed using descriptive statistics (i.e., frequencies and means). Qualitative data from the open-ended questions were used to support and add depth to the quantitative findings. As such, qualitative data were divided deductively under two pre-determined headings namely “encouraging reactions” and “discouraging reactions.” Illustrative participant excerpts were used to support these findings.

## Results

A total of 130 surveys were completed. Of those, 22 were completed by Cohort 1 (secondary school male students) and 108 were completed by Cohort 2 (male nurses and nursing students).

### Influencing factors

**Fig 1:**
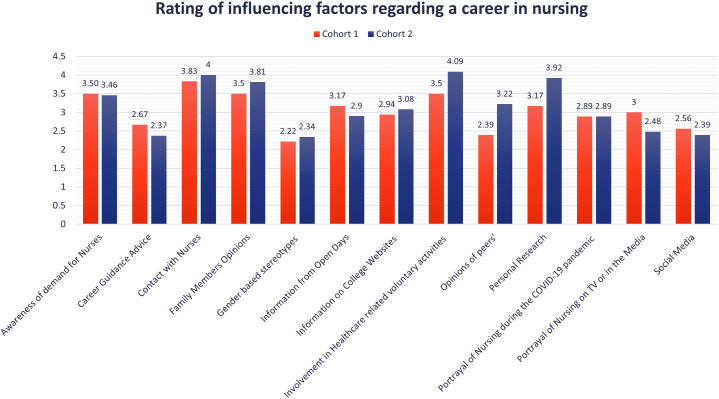
Mean scores of influencing factors for Cohort 1 (secondary school students) and Cohort 2 (nurses and nursing students) out of a maximum possible score of 5.

Participants from both cohorts were presented with a series of questions which sought to determine the extent to which particular factors impacted their thinking (Cohort 1) or decision (Cohort 2) regarding a career in nursing (Fig 1). Cohort 1 ranked the opinions of their peers (mean 2.39/5) and gender-based stereotypes (mean 2.22/5) as having the least impact on their decision. Of note, these are typically referred to as influential factors in dissuading men from pursuing a career in nursing [9]. The men in Cohort 2 who had already chosen nursing, appear to have agreed with this rating (mean 2.34/5) as they also reported gender-based stereotypes as the least impactful on their career decisions.

### Ranking the influencing factors

The most influential factors according to Cohort 1 (secondary school male students), were previous contact with nurses, the opinions of their families, an awareness of demand for nurses, and involvement in healthcare-related voluntary activities. Cohort 2 also ranked involvement in healthcare related voluntary activities, previous contact with nurses, such as having previously worked alongside nurses as a healthcare assistant, as having had the biggest impact on their decision to pursue a career in nursing. Both cohorts ranked advice from career guidance/counselling as having very little impact which is congruent with the findings in some of the literature [11]. The media portrayal of nursing, as well as social media, were ranked quite low by both cohorts, which contradicts findings elsewhere [13,15].

### Reactions to choosing nursing as a career

**Fig 2:**
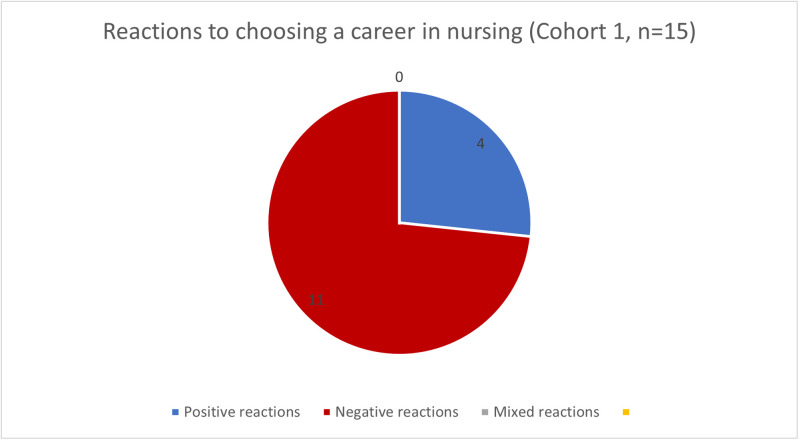
Cohort 1 (secondary school students) analysis of responses to “What reactions have you noted from your friends and family when you chose a career in nursing?”.

**Fig 3:**
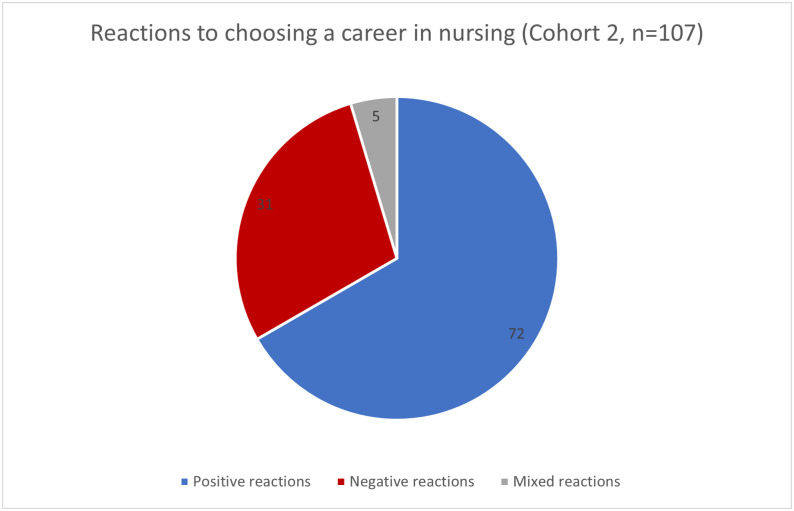
Cohort 2 (nurses and nursing students) analysis of responses to “What reactions have you noted from your friends and family when you chose a career in nursing?”.

While these did appear to range between positive and negative, some of the negative reactions were quite remarkable. Fifteen of 22 participants in Cohort 1 (secondary school students) answered this question. These participants received proportionately more negative reactions (See Fig 2) when discussing a career in nursing in comparison to Cohort 2. Of note, there is a significant difference in the sample sizes between Cohorts 1 and 2. Of the 15 responses provided by Cohort 1, 73.3% (n = 11) said they received a negative reaction from friends or family members when discussing choosing nursing. All 108 participants within Cohort 2 answered this question. They recalled more positive reactions to their decisions with 66.7% (n = 72) reporting positive reactions and 4.6% (n = 5) reporting mixed reactions (See Fig 3).

It is noteworthy that participants gave very detailed, considered, and enthusiastic contributions in the qualitative free-text sections of the survey. Many commented that they were glad to see this issue being researched. In order to gain some in-depth qualitative insights, responses to open-ended questions were categorised into “encouraging reactions” and “discouraging reactions.”

### Encouraging reactions

Some respondents in Cohort 1 (secondary school male students) received positive and encouraging feedback. For example, respondent 10 told of how his friends and family were very proud of his decision, stating: *“They think it’s a great career choice. Very proud of me.”*

Reactions from friends and peers were overwhelmingly positive for Cohort 2 (nurses and nursing students). Respondent 3 told of how his family saw it as a means to help people and were happy with his decision: “*They were very happy. They viewed it as a way in which I could help people. Gender was never an issue or a topic that ever came up as my friends and family don’t subscribe to gendered roles for jobs etc*.” Other responses echoed similar reactions of positivity and encouragement. Respondent 12 noted similar feelings of “*overwhelming support for my decision to work as a nurse.*”

Many respondents told of how their families believed nursing to be a perfect job for them as it matched their personalities. For example, respondent 86 in Cohort 2 spoke of how his friends and family regarded him as perfect for the job: “*My family and friends were always encouraging, saying I had the right personality for nursing. I have a few friends and family in nursing, and they were supportive of my decision. My family are proud that I am a nurse now.”* Another respondent, while positive, brought in the issue of gendered roles stating: “*Vast majority of my family have always been very supportive & have noted that Nursing is a career that fits my personality & skills. However, at times it’s mentioned that I should have considered ‘joining the fire brigade’ or a similar area of male dominated activities.*”

### Discouraging reactions

Cohort 1 appeared to face more negative commentary from parents and friends regarding their decision. One respondent told of a reaction of “*Horror, amazement, disgust, surprise, disappointment, contempt,*”. Other reactions were equally as negative, with one respondent saying that his *“family are supportive but sceptic. Friends were very unsupportive.”* Other negative reactions voiced by respondents related to the topic of pay, and the view of nursing as a “lesser” medical field. Respondent 8 described his family’s negative response in relation to the pay, stating: *“The pay is abysmal, and I could pick a “better” medical field.”* It appears overall that most respondents from Cohort 1, those who had not yet chosen their career, were being steered away from choosing nursing.

Sexuality and gender stereotyping did not emerge as an issue of concern for most of our participants. Indeed, only three of the 108 Cohort 2 respondents referenced reactions to nursing as being a female profession and/or associated with homosexuality. One man commented “*A lot of negative reactions from family and friends. One or two close friends were more supportive. I was asked if I was gay/homosexual by a family member*” (Respondent 54). A similar reaction was reported by respondent 51 who said: “*All, and I mean all, smirked or laughed at the fact I wanted to be a nurse especially my friends* […] *mocking me saying* […] *that I was “gay” for picking nursing, that it’s acceptable for a gay man to be a nurse and not a straight man.”*

## Discussion

This study captured the views of men in nursing and explored the opinions of secondary school students to understand their decision-making regarding nursing as a career choice, and the factors that influence those decisions. The impact of gender stereotyping, and its impact on men in the nursing profession has been widely studied and documented in other papers [21]. It would appear that, to the general public, nursing is often seen as “lesser” or “lower” than other medical professions or fields such as medicine. This had the effect of turning men off the profession or acting as a deterrent for those already in the profession or studying nursing [18]. Some have suggested this may be due to the fact that men may like to be seen as the breadwinners and this perception of nursing, as a “lesser” field may not help this idea [12].

Findings from our study did not appear to agree with these studies. In fact, we found that gender-based stereotypes ranked as the least impactful for both Cohort 1 (mean 2.22/5) and Cohort 2 (mean 2.34/5) in terms of acting as an influencer in their decision to choose nursing. This could be due to the fact that men entering into nursing is being seen as increasingly normal, and as such, the stereotype of nursing as a woman’s career is perhaps dissipating. Of note, these scores were derived from our instrument, whereby respondents ranked a number of pre-determined factors as being “Least Influential” to “Most Influential” on a five-point Likert scale. This finding suggests that despite being exposed to gender stereotypical criticisms of their career choice, as seen in Powers et al. [13] and Yang et al. [22], men in our study did not appear to have allowed the “prevailing definitions of masculinity” to have “acted as a powerful barrier to men crossing the gender divide and entering the profession” [23] (p. 321). It could be hypothesised that the men in our study were enabled to look beyond the superficiality of gender stereotyping, resulting in the lack of impact contrasting with discussions in other studies [9,21].

Our findings also provide a contrast to much of the evidence regarding media portrayals of men in nursing. It is widely regarded that such portrayals are influential [13](Powers et al., 2018), and that “such images can have potentially negative implications for recruitment practice and the profession” (p.389) [24]. Perhaps, our respondents failed to rank social media portrayals as having been particularly influential in their decisions to choose a career in nursing (Cohort 1: mean 2.56/5 and Cohort 2: mean 2.39/5). Portrayals of nursing on television or in media were slightly more influential (mean 3/5 and mean 2.48/5 respectively), indicating less of a focus on social media than might have been assumed.

The fact that respondents in our study ranked “contact with nurses and involvement in healthcare related/voluntary activities” as the highest impacting factor provides an important message to the profession. Our findings confirm those of other studies that have emphasised the importance of encouragement from other nurses and male role models [13]. Yi and Keogh [25], for example, in their systematic review of the motivations of men who choose nursing as a profession, also identified that early exposure to nurses and other health professionals was significantly influential. If experiences with healthcare and with nurses are important, it therefore falls to all nurses to be conscious of how they nurture this. Each nurse bears an important responsibility of portraying an image of nursing that is inclusive and welcoming of diversity.

Arguments for diversity usually involve confronting unequal power relations and calling upon those in a majority to welcome the “other” who find themselves in the minority and experiencing discrimination. Arguments for greater diversity usually bring with them an act of advocacy for the voiceless and the vulnerable [26]. However, any suggestion of vulnerability is undermined by evidence of a disproportionate number of men holding senior positions in management and in academia [27–29]. It is therefore seen by some as inappropriate to locate the argument for diversity in nursing as one that involves advocacy for an underrepresented group. In response to this, it should be argued that a more diverse nursing workforce offers a stronger and more resilient base upon which to make the case for better recognition, remuneration, and conditions for the profession. Complexities, however, exist. For example, research from the United States of America found a significant gender-related pay gap among Nurse Practitioners, which was in line with the findings of pay gaps within other female dominated professions [30]. That we should have to recruit more men into nursing to achieve this is understandably a source of frustration and contention for many, particularly those examining the issue from a feminist perspective. Consequently, the encouragement of more men into a career in nursing is not something necessarily embraced by all. At a time of a global shortage of nurses of over 4 million [31], all possible efforts should be made to increase the nursing workforce. Encouraging more men into nursing will, in and of itself, contribute to this. Additionally, male nurses’ potential impact on pay and working conditions should not be underestimated in terms of enhancing the attractiveness of a nursing career.

### Implications

This study sheds light on the potential barriers to men considering entering a nursing career. As such, by identifying these barriers, we may begin to break them down, thus making the field of nursing more accessible and inclusive. Findings from this study can be used to help schools of nursing target their advertisement and outreach. This study also allows us to hear from the minority within the field and helps challenge several preconceived barriers for men entering nursing. Our study has shown that media portrayals and social media may have little bearing on men’s choices to pursue a career in nursing, when compared to family influences or previous contacts with nurses and healthcare professionals.

From a research perspective, sample size calculation is warranted in future research to enhance the generalisability of findings. While the present study had no theoretical underpinning, there is indeed scope to include a theoretical framework/model in future research in this area. Moreover, future exploratory studies (e.g., qualitative studies) would provide more in-depth insights into factors that influence men’s decisions to choose nursing as a career. Future research also ought to explore the potential societal impact of this research on secondary school students who are considering nursing as a career.

### Limitations

There are some limitations to our study that should be acknowledged. Cohort 1 was restricted to over 18-year-olds, an age at which many career choices have already been made. It would be worthwhile exploring the views of young men in the younger age range so as to see how a nursing career choice is viewed by teenagers. Another limitation includes the fact that Cohort 2 were recalling their experiences and their recollection of the factors that influenced their decisions may have been influenced by the passage of time and by their preferred rationale for choosing a career in nursing. Another limitation relates to the relatively small sample size, the relative sample size difference between the two Cohorts, and the small sample size of Cohort 1. This was partly due to the use of convenience and snowball sampling strategies, as well as difficulty with recruiting secondary school students during summer months. While social media metrics (e.g., engagements and impressions) were captured, it was not possible to determine how many of these engagements resulted in participation. Therefore, an exact response rate could not be calculated.

## Conclusions

This study provides valuable findings regarding men’s decision-making around a career in nursing. Assumptions surrounding the factors that impact on this career choice should be reviewed regularly and specific investments in recruitment campaigns and their resources should be tailored with these in mind. Resources being invested in specific guidance and social media campaigns may be less influential than anticipated. Assumptions that gender stereotypes and assumptions about masculinity need to be continually challenged as they become outdated. A key finding from our study is the import which men placed on their previous experience in voluntary health related areas, such as First Aid, and the equally important impact of their interactions with nurses in shaping their career decision. Encouraging men into nursing is therefore every nurse’s responsibility.

## Supporting information

S1 File Survey Questions. (DOCX)
