# Supplementary material for: Factors influencing men’s decisions about a career in nursing
Source: PLoS One. 2025 Dec 5;20(12):e0337360. doi: 10.1371/journal.pone.0337360 (PMC12680186; doi:10.1371/journal.pone.0337360)
Supplement: S1 File — (DOCX) [file pone.0337360.s001.docx]

**SUPPLEMENTAL FILE 1: SURVEY QUESTIONS**

**COHORT 1 (Men in Secondary Schools):**

**Five-point Likert Scale, ranging from 1 star for “Very Discouraging” to 5 stars for “Very Encouraging”:**

1. Have you considered choosing a career in Nursing?
2. What type of second level school do you/did you attend? (A boys’ school or a co-ed)
3. How would FAMILY MEMBERS' OPINIONS impact your thinking about a career in nursing?
4. How would your CAREER GUIDANCE ADVICE impact your thinking about a career in nursing?
5. How would INFORMATION FROM OPEN DAYS impact your thinking about a career in nursing?
6. How would INFORMATION ON COLLEGE WEBSITES impact your thinking about a career in nursing?
7. How would CONTACT WITH NURSES (e.g. as a patient or a relative of a patient) impact your thinking about a career in nursing?
8. How would PORTRAYALS OF NURSING ON TV OR IN MOVIES impact your thinking about a career in nursing?
9. How would INVOLVEMENT IN HEALTH CARE RELATED VOLUNTARY ACTIVITIES (e.g. First Aid) impact your thinking about a career in nursing?
10. How would your PEERS' OPINION impact your thinking about a career in nursing?
11. How would findings from your own PERSONAL RESEARCH impact your thinking about a career in nursing?
12. How would your AWARENESS OF DEMAND for nurses impact your thinking about a career in nursing?
13. How would PORTRAYALS OF NURSING DURING THE COVID19 PANDEMIC impact your thinking about a career in nursing?
14. How would SOCIAL MEDIA impact your thinking about a career in nursing?
15. How would GENDER BASED STEREOTYPES impact your thinking about a career in nursing?

**Open-ended Questions:**

1. Any other influencing factors that we have not included here. Please tell us more about any factors influencing your career choice and how influential they were.
2. What reactions have you noted from your friends and family when discussing a potential career in nursing?
3. In your opinion what would be achieved by encouraging more men into careers in nursing?
4. If you have considered a career in nursing, in which area of nursing do you think you would be interested?
5. Are there any other comments you would like to make about the topic of men in nursing?

**COHORT 2 (Men already in Nursing/Student Nurses):**

**Five-point Likert Scale, ranging from 1 star for “Very Discouraging” to 5 stars for “Very Encouraging”:**

1. How did the opinions of FAMILY MEMBERS impact your decision to choose a career in nursing?
2. How did your CAREER GUIDANCE ADVICE impact your decision to choose a career in nursing?
3. How did INFORMATION FROM OPEN DAYS impact your decision to choose a career in nursing?
4. How did INFORMATION ON COLLEGE WEBSITES impact your decision to choose a career in nursing?
5. How did CONTACT WITH NURSES (e.g., as a patient or a relative of a patient) impact your decision to choose a career in nursing?
6. How did PORTRAYALS OF NURSING ON TV OR IN MOVIES impact your decision to choose a career in nursing?
7. How did INVOLVEMENT IN HEALTH CARE RELATED VOLUNTARY ACTIVITY (e.g., First Aid) impact your decision to choose a career in nursing?
8. How did your PEERS' OPINIONS impact your decision to choose a career in nursing?
9. How did findings from PERSONAL RESEARCH impact your decision to choose a career in nursing?
10. How did your AWARENESS OF DEMAND for nurses impact your decision to choose a career in nursing?
11. How did PORTRAYALS OF NURSING DURING THE COVID19 PANDEMIC impact your decision to choose a career in nursing?
12. How did SOCIAL MEDIA impact your decision to choose a career in nursing?
13. How did GENDER BASED STEREOTYPES impact your decision to choose a career in nursing?

**Open-ended Questions:**

1. Any other influencing factors that we have not included here?
2. What reactions have you noted from your friends and family when you chose a career in nursing?
3. In your opinion what would be achieved by encouraging more men into careers in nursing?
4. In which area of nursing do you work?
5. How many years have you been in nursing
6. Are there any other comments you would like to make about the topic of men in nursing?
